# Supplementary material for: Comparison between the Effects of Adding Vitamins, Trace Elements, and Nanoparticles to SHOTOR Extender on the Cryopreservation of Dromedary Camel Epididymal Spermatozoa
Source: Animals (Basel). 2020 Jan 2;10(1):78. doi: 10.3390/ani10010078 (PMC7022978; doi:10.3390/ani10010078)
Supplement: Supplementary file 1 [file animals-10-00078-s001.pdf]

Table S1. Characteristics of ZnONPs and SeNPs.

Sample	Particle size (nm)	PDI	Zeta potential (mV)
ZnONPs	30.92 ± 1.25	0.54 ± 0.006	32.16 ± 0.252
SeNPs	78.47 ± 17.93	0.29 ± 0.23	-20.36 ± 1.79

DPI: polydispersity index

Figure S1. Representative particle size distribution (A) and zeta potential (B) curves of ZnNPs.

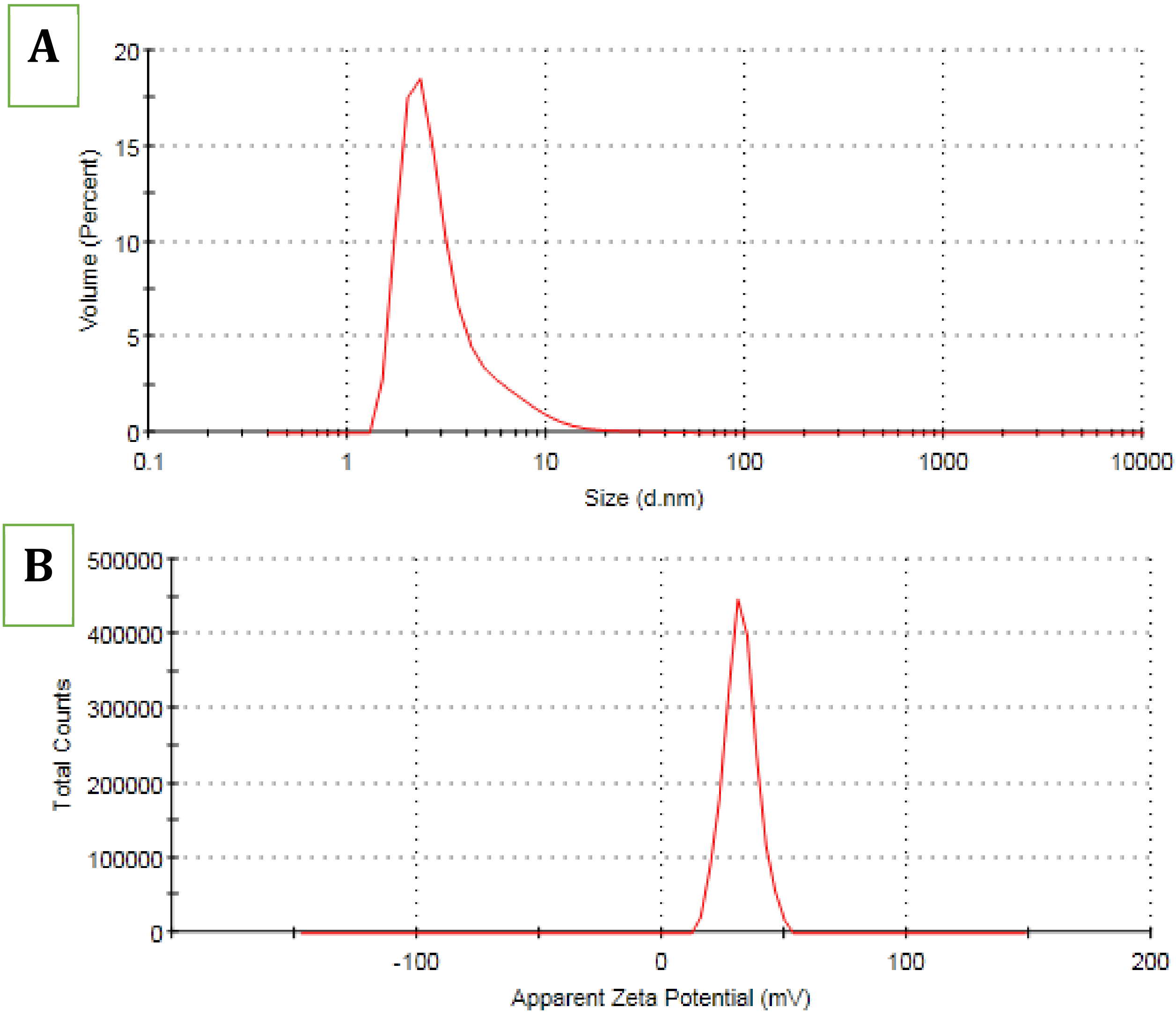

Figure S2. Representative particle size distribution (A) and zeta potential (B) curves of SeNPs.

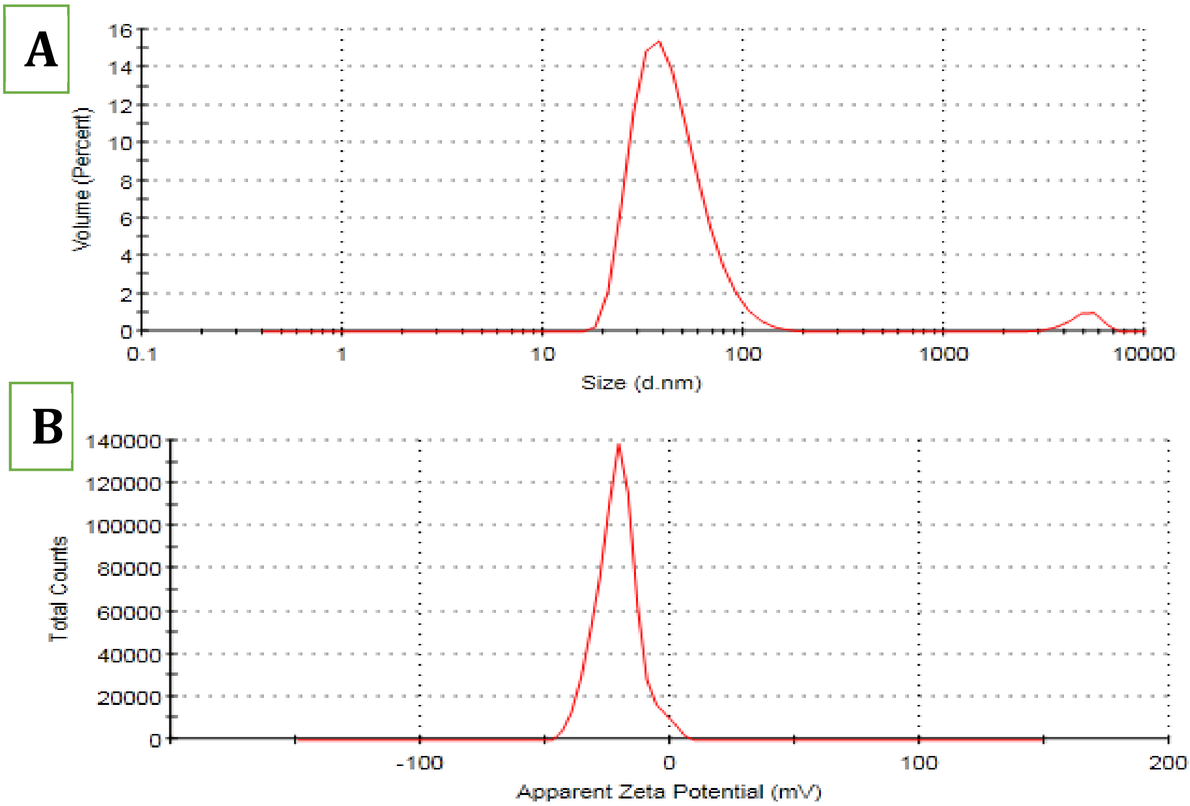

Figure S3. The raw data of two replicates for annexin V flow cytometry.

<table>
<tr><th>R1</th><th></th><th>R2</th></tr>
<tr><td colspan="3">Control</td></tr>
<tr><td>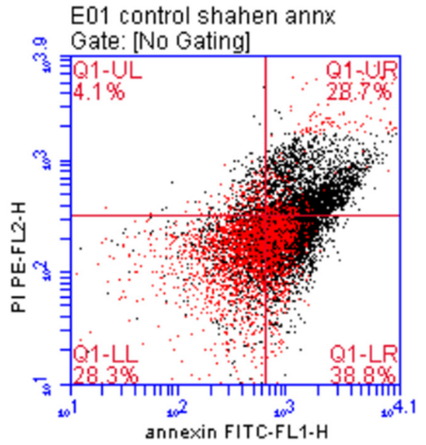
</td><td></td><td>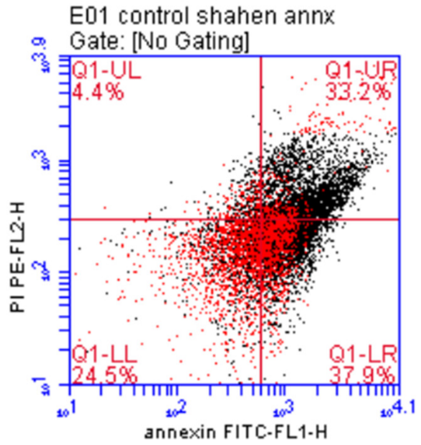
</td></tr>
<tr><td colspan="3">Vit C</td></tr>
<tr><td>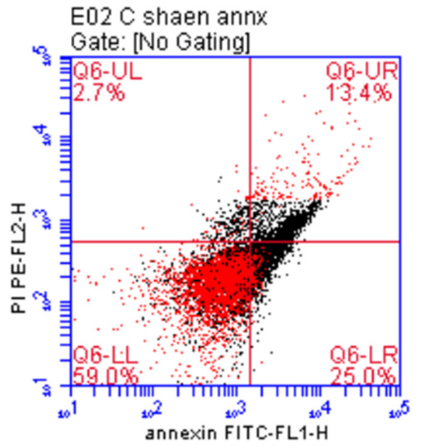
</td><td></td><td>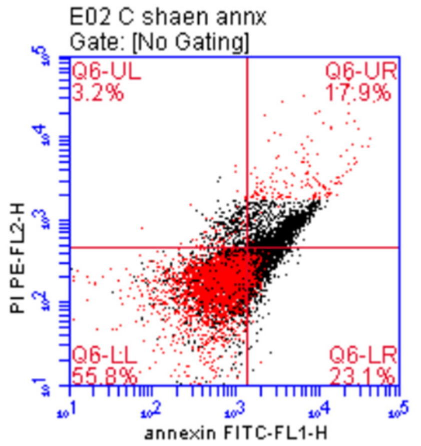
</td></tr>
<tr><td colspan="3">Vit E</td></tr>
</table>

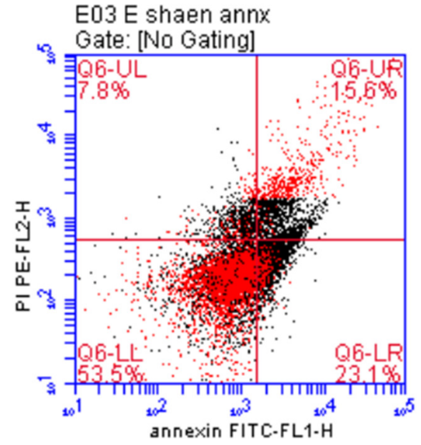

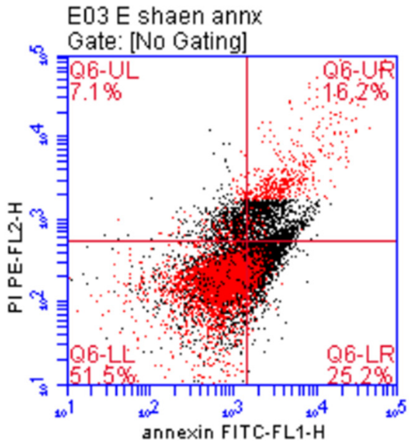

Nano Se

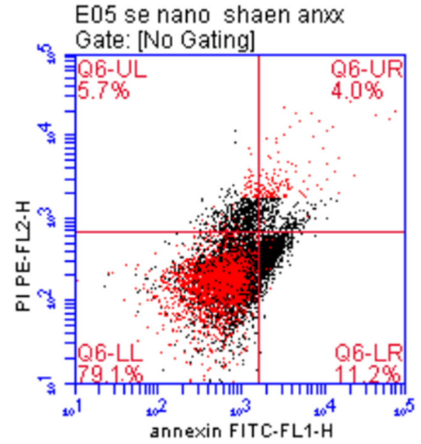

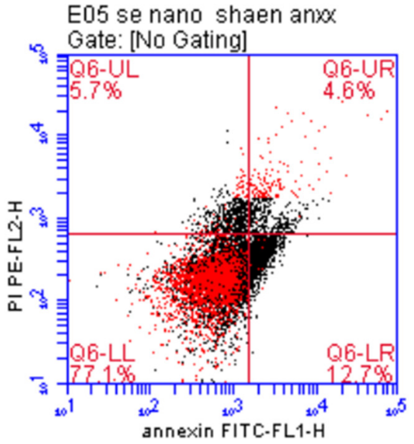

Sodium selinate

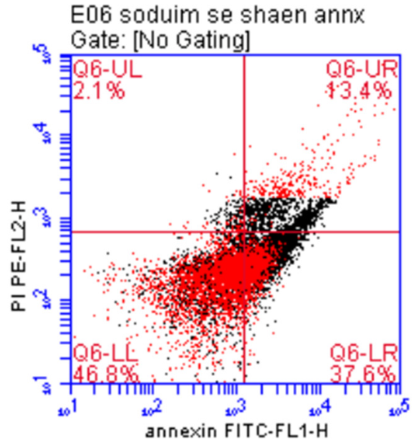		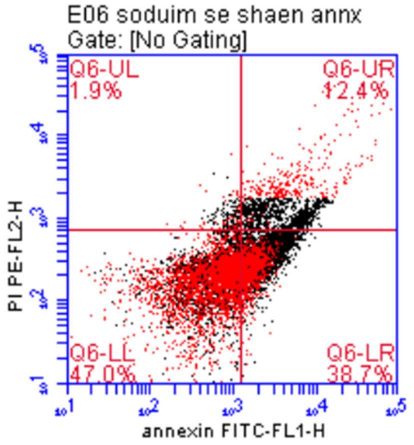
**Nano Zn**		
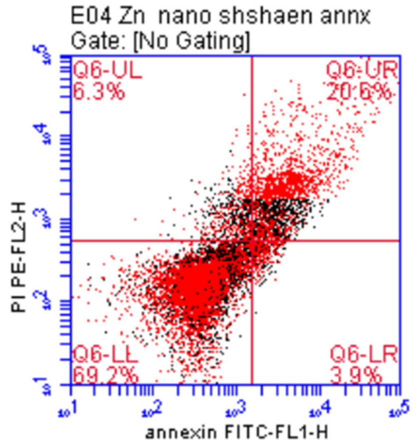		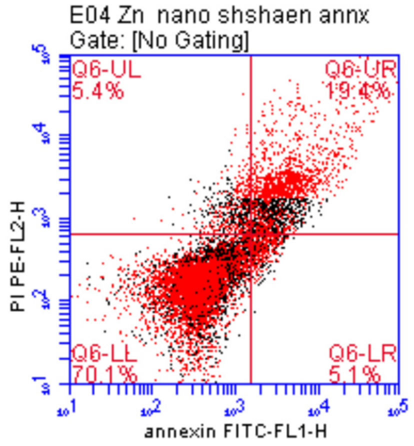
**Zn sulphate**		

<table>
<tr>
<td>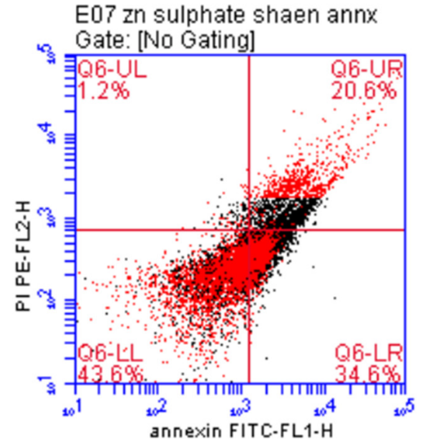
</td>
<td></td>
<td>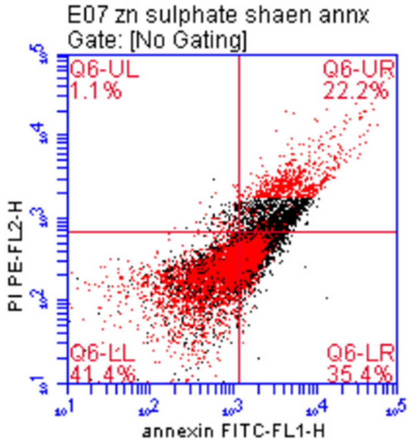
</td>
</tr>
<tr>
<td></td>
<td></td>
<td></td>
</tr>
</table>
